# Cord blood-derived iNK T cells as a platform for allogeneic CAR T cell therapy

**DOI:** 10.3389/fimmu.2025.1621260

**Published:** 2025-05-30

**Authors:** Maison Grefe, Abel Trujillo-Ocampo, Jelita Clinton, Hong He, Ling Yu, Dan Li, Qing Ma, Elizabeth J. Shpall, Jeffrey J. Molldrem, Jin S. Im

**Affiliations:** ^1^ Department of Hematopoietic Biology & Malignancies, The University of Texas MD Anderson Cancer Center, Houston, TX, United States; ^2^ Department of Stem Cell Transplantation & Cellular Therapy, The University of Texas MD Anderson Cancer Center, Houston, TX, United States

**Keywords:** invariant natural killer t cells, cord blood, chimeric antigen receptor, acute myeloid leukemia, CAR T cell therapy

## Abstract

CD1d-restricted invariant Natural Killer (iNK) T cells are a suitable candidate for allogeneic Chimeric Antigen Receptor (CAR) T cell therapy as they do not cause graft-versus-host disease (GvHD) due to the monomorphic nature of CD1d proteins. However, the phenotypic and functional heterogeneity of iNK T cells from adult donors (AD) may lead to the inconstant CAR-iNK T cell products. Cord blood-derived (CB) iNK T cells, in contrast, exhibit inter-donor homogeneity in phenotype including uniform CD4 expression and are enriched in memory iNK T cell populations. Thus, we evaluated the preclinical therapeutic potential of iNK T cells derived from cord blood (CB) as an off the shelf CAR T cell therapy platform, given the dominant presence of CD4^+^ iNK T cells. First, CB-derived iNK T cells were extremely enriched with CD4^+^ iNK T cells that express various NK receptors and display iNK-TCR mediated cytotoxicity but in a lesser degree than AD-derived CD4^-^ iNK T cells. When engineered with an 8F4CAR targeting the acute myeloid leukemia-associated antigen PR1 presented in HLA-A2*01, CB-8F4CAR-iNK T cells showed a greater expansion capacity with higher CD62L expression than AD-8F4CAR-iNK T cells but with similar 8F4CAR expression and iNK T purity. CB-8F4CAR-iNK T cells displayed *in vitro* cytotoxicity against PR1/HLA-A2^+^ primary Acute Myeloid Leukemia (AML) and cell lines better than AD-8F4CAR iNK T cells and maintained potent cytotoxicity in repeated antigenic challenges. Moreover, CB-8F4CAR-iNK T cells showed anti-leukemia activity *in vivo* in a dose dependent manner. Lastly, CB-8F4CAR-iNK T cells were polarized to produce Th2-biased cytokines but in a lesser amount after 8F4CAR-mediated leukemia cytolysis compared to iNK-TCR mediated activation. In conclusion, consistent CD4^+^ phenotype, superior expansion capacity, and enhanced CD62L expression of CB-CAR-iNK T cells suggest that they may provide an alternative off-the-shelf source for effective CAR-iNK T cell therapy, while reducing the risk of severe cytokine release syndrome through their immunomodulatory properties. Thus, our results support the potential use of CB-iNK T cells as an allogeneic CAR-T cell therapy platform as they maintain a potent cytotoxicity with potentially better safety profile given a Th2-biased cytokine production upon activation.

## Introduction

The emergence of cancer immunotherapy has opened new avenues for the treatment of cancer. Chimeric antigen receptor (CAR) T cell therapy has shown remarkable success in treating certain hematological malignancies (1, 2), but still faces significant challenges, including the lack of a uniformly expressed, cancer-specific antigen, the risk of severe on-target off-tumor toxicities, and cytokine release syndromes (3–5). Additionally, the autologous CAR-T cell product may have limitations such as the need for autologous T cells, high production costs, additional time to produce, and potential manufacturing failures (6). Off the shelf, allogeneic CAR-T cells can potentially overcome such short-comings of autologous CAR-T cells but graft-versus-host disease (GvHD), inadequate *in vivo* persistency, or rejection are challenges that remain to be addressed (7).

The invariant Natural Killer (iNK) T cells offer several compelling advantages as an off the shelf CAR platform, making them an attractive alternative to conventional αβ T cells. Unlike αβ T cells, iNK T cells do not cause GvHD in allogeneic settings due to their restriction to the monomorphic CD1d protein (8, 9). This unique feature allows for the development of “off-the-shelf” allogeneic therapies, potentially reducing production costs and treatment delays. Furthermore, iNK T cells possess inherent anti-cancer mechanisms that complement CAR-mediated activity. They express natural killer cell activating receptors for additional synergistic effects, eliminate tumor-associated immunosuppressive macrophages, reprogram favorable tumor immune-microenvironment, and enhance adaptive ant-tumor immunity via CD40-CD40L feedback loop (10–12). Additionally, iNK T cells demonstrate superior tumor infiltration capabilities compared to αβ T cells, particularly in solid tumors, which could broaden their therapeutic applications beyond hematological malignancies (13). These combined attributes position iNK T cells as a promising platform for next-generation CAR therapies. Indeed, interim analysis of a clinical trial with patients treated with allogeneic AD-CD19CAR-iNK T cells show excellent safety profile, including no grade 3 or higher toxicities, with high rates of objective response (NCT00840853) (14).

While AD-CAR-iNK T cells appear promising, they are well documented to be heterogeneous in phenotype and functional activity (15–17). For example, iNK T cells express CD4, CD8α, or are double-negative (DN), and these markers largely determine the cytokine production profile of stimulated iNK T cells where CD4^+^ cells are TH2-polarized and CD8α^+^ cells are TH1-polarized (18). This heterogeneity potentially complicates optimal healthy donor iNK T cell selection and could result in inconsistent efficacy of the CAR-iNK T product between manufacturing runs. In contrast, cord blood-derived (CB-) iNK T cells display uniformity in CD4 expression with >90% being CD4^+^, which suggests inter-donor consistency in iNK T cell function (19, 20). In this study, we investigated the potential use of CB-iNK T cells as a platform for off the shelf CAR T cell therapy.

## Methods

### Materials

All research was conducted in accordance with the Declaration of Helsinki and The University of Texas MD Anderson Cancer Center Institutional Review Board guidelines. Buffy coats were purchased from MD Anderson Blood Bank, and deidentified cord blood units for general translational research were provided by MD Anderson Cord Blood Bank. Deidentified primary AML cells were provided by the Departmental Tissue Bank at MD Anderson Cancer Center. All animal experiments were performed under the University of Texas Institutional Animal Care and Use Committee (IACUC)-approved protocols.

Wild-type U937 and K562 cell lines were originally acquired from ATCC and transduced with HLA-A2 or CD1d, and/or additional GFP. All cells were cultured in complete media (RPMI 1640 [Gibco 11875093], 10% heat-inactivated FBS [Gibco #A5256701], 1mM Glutamine [Gibco #25030081], 50uM Non-essential Amino Acids [Gibco #11140050], 25uM Essential Amino Acids [Gibco #11130051], 10uM HEPES [Gibco #15630080], 10ug/mL gentamicin [Gibco #15710064], 50uM β-mercaptoethanol [Gibco #21985023]) and confirmed to be mycoplasma negative prior to experiments.

### Retrovirus production

Retrovirus containing 8F4CAR was produced by co-transfecting 293GP cells with RD114 envelope coding plasmid along with 8F4CAR in pSFG vector using the lipofectamine 2000 system (Thermo Fisher #11668019) according to manufacturer’s protocol. In short, 293GP cells were seeded in 10cm Poly-D-Lysine coated plates 16 hours prior to transfection. Media was gently replaced as to not disturb the cells before transfection. Then in 1.5mL OPTI-MEM media (Thermo Fisher #31985070) 9ug of 8F4CAR plasmid was mixed with 4.5ug RD114 plasmid in one conical tube; in another conical tube 60uL of lipofectamine 2000 was mixed in 1.5mL OPTI-MEM. After 5 mins the two solutions were gently mixed and plasmid-lipofectamine complexes were allowed to form for 20 mins prior to dropwise addition to 293GP cells. After 48–72 hrs, retrovirus-containing supernatant was collected and spun at 2000g to remove cell debris before aliquoting and storing at -80C. Retroviral titer was determined with QuickTiter Retrovirus Quantitation Kit by Cell Biolabs Inc. (VPK-120) as per manufacturer instructions.

### Primary iNK T cell isolation, activation, and 8F4CAR transduction

Mononuclear cells were isolated by Histopaque (Sigma #10771) density gradient centrifugation, followed by iNK T cell enrichment with anti-iNK T micro-beads according to manufacturer’s protocol (Milyteni Biotec #130-094-842). Enriched iNK T cells were split in half to generate donor-matched UT- and 8F4CAR-iNK T cells and stimulated with irradiated allogeneic DCs in complete media that were plated and pulsed with 100nM α-Galactosylceramide (αGalCer) (Avanti #867000) one day prior. After 3 days of stimulation in the presence of 50IU/mL recombinant human IL-2 (Peprotech #200-02), iNK T cells were transduced with retrovirus harboring 8F4CAR retrovirus as previously reported (21). Briefly, one day prior to transduction, 24 well non-tissue culture treated plates (Thermo #0877251) were coated with RetroNectin (Takara #T100A) at 7ug/mL in sterile PBS (Thermo #J61196-AP) for 16hrs at 4C. On the day of iNK T cell transduction, the RetroNectin treated plates were removed from 4C and brought to room temperature. Then the RetroNectin solution was aspirated, and complete media was added for 30 mins in 37C before media was replaced with 1mL retrovirus-containing supernatant. The plate was then wrapped in parafilm and centrifuged at 2000g for 2 hr at 32C. The viral supernatant was removed and stimulated iNK T cells were added in complete media supplemented with 50IU/mL IL-2. The iNK T cells were cultured for 14 days and re-stimulated with αGalCer pulsed allogeneic DCs at a ratio of 4 iNKT cells to 1 dendritic cells in 24 well plate (2x10e6 iNKT cells/well) or in G-rex6 well plate (upto 10x10e6 iNKT cells/well, Wilson Wolf,Manufacturing, MN) for an additional 14 days before the final count and phenotype was assessed by flow cytometry. Cells were frozen and stored in liquid nitrogen for further functional analysis.

### Flow cytometry

For phenotypic assessment, iNK T cells were stained with various fluorochrome conjugated antibodies at 1ug/ml in PBS and covered from light for 15 mins at room temperature. Cells were then washed with PBS, labeled with live/dead cell dye (BD #564996), washed again with PBS, and resuspended in PBS for acquisition on the LSRFortessa X-20 Cell Analyzer. For intracellular cytokine staining, 8F4CAR-iNK T cells were stimulated with target leukemia cells or αGalCer-pulsed dendritic cells at 1:1 E:T ratio for 8 hours, or 30ng/mL PMA (Sigma #P8139) and 1ug/mL Ionomycin (Sigma #I0634) as a positive control, in the presence of protein transport inhibitors Brefeldin A (BD #555029) and Monensin (BD #554724). Cells were then washed with PBS and stained for surface markers as described above before fixation and permeabilization with 1X Fix/Perm buffer (BD #554714). Cells were washed with 1X Perm/Wash buffer (BD #554714) and resuspended in intracellular cytokine antibody solution (in 1X Perm/Wash buffer) for 30 mins at room temperature. Lastly, cells were washed with 1X Perm/Wash buffer and resuspended in PBS for immediate acquisition on the BD LSRFortessa X-20 or the Cytex Aurora cytometer. Analysis was performed using FlowJo v10.8. Antibody-Fluorochrome conjugates against following antigens were purchased from BD Biosicence, biolegend, R&D Systems, and Thermofisher: CD3-PE (clone SP34-2), CD3-PerCP (Clone SK7), CD3-FITC (Clone HIT3a), CD4-BUV395 (Clone SK3), CD8a-BV510 (Clone SK1), iNKT-PE (Clone 6B11), iNKT-PE-Cy7 (Clone 6B11), CD45RA-BV421 (Clone HI100), CD62L-BV786 (Clone SK11), IgG-APC (Polyclonal), IFNγ-APC-Cy7 (Clone B27), IL-4-PE-Cy7 (Clone 8D4-8), TIM3-BV711 (Clone 7D3), LAG3-BV605 (Clone T47-530), PD1-BV786 (Clone EH12.1), CTLA4-PE-Cy7 (Clone BNI3), CD69-BV510 (Clone FN50), ICOS-BUV737 (Clone DX29), GITR-APC-eFluor780 (Clone DTA-1), CD27-PECF594 (Clone O323).

### Cytotoxicity assay

Effector iNK T cells were thawed and rested in complete media for 16 hrs prior to functional assessment. Target cell lines were cultured for 1 week before use in cytotoxicity assays, and primary leukemia samples were thawed one day prior and rested in complete media. Target cell lines were stained in the dark with 1uM Cell Trace Violet (Thermo #C34557) in PBS at 37C for 20 mins before quenching and washing with 5X volume complete media. Primary leukemia target cells were not labeled with cell trace dye to preserve viability and instead were identified by CD33 and CD34 staining. Target cells were plated at 10K cells/well in triplicates in 96 well round bottom plates (Corning #3799) before addition of effector cells at the according E:T ratios. The co-culture was carried out for 16 hrs before cells were washed with PBS, stained with live dead cell dye (and CD33 and CD34 in the case of primary leukemia), counting beads were added (Sigma #C36950), and cells were acquired on the LSRFortessa X-20. Percent specific killing was calculated: [(experimental number live target cells – number live target cells alone in media)/(0 – number live target cells alone in media)] x 100.

### Serial challenge assay

U937 HLA/A2^+^ GFP^+^ cells were co-cultured with effector cells at a 1:1 E:T ratio at 50K iNK T cells in triplicates in 96 well round bottom plates in complete media supplemented with 50 IU/mL IL-2. On the same day of experiment set-up, a sample was washed with PBS, stained with Live/Dead cell dye, and counting beads were added to establish the initial absolute number of effector and target cells. Every 3–4 days a sample was taken and counts of target and effector cells were recorded. On the day of acquisition, new U937 HLA/A2 GFP target cells were added to maintain an E:T ratio of 1:1 in fresh complete media supplemented with 50 IU/mL IL-2.

### Cytokine production analysis by ELISA

iNK T cells were co-cultured with wild-type U937 or HLA/A2 U937 cells for 24 hours in a 1:1 E:T ratio at 50K iNK T cells per well in triplicates. Supernatant was collected and analyzed for the presence of IFNγ, IL-4, and IL-10 using BD capture and detection antibody pairs in 96 well high-binding plates (Corning #3690). Streptavidin-HRP was added, and luminescence signal was read on the Cytation 3 (Fisher Scientific). Following antibodies and reagents were purchased from BD bioscience Anti-human IFNγ-Capture (Clone NIB42), Anti-human Biotin-IFNγ-Detection (Clone 4S.B3), Recombinant human IFNγ-Standard, Anti-human IL-4-Capture (Clone 8D4-8), Anti-human Biotin-IL-4-Detection (Clone MP4-25D2), Recombinant human IL-4-Standard, Anti-human IL-10-Capture (Clone JES3-9D7), Anti-human Biotin-IL-10-Detection (Clone JES3-12G8), Recombinant human IL-10-Standard, Streptavidin-HRP.

### Xenogeneic mouse model

Female 6–8 week old NSG (NOD.Cg-Prkdc<scid>Il2rg<tm1Wjl>/SzJ) mice were obtained from the Jackson Laboratory. NSG mice between the ages of 8 and 12 weeks were irradiated with 200 cGy using the Cesium-137 irradiator on day -1 and were intravenously (i.v.) injected with 5.0 x 10^3^ U937 HLA-A2^+^ GFP/Luc^+^ cells on day 0. Three days later, treatment cells were administered i.v. at the designated doses. Leukemia progression was monitored by BLI imaging on the IVIS Lumina X5 (PerkinElmer) at the MDACC Small Animal Imaging Facility (Smith Research Building) once weekly. Moribund mice were sacrificed per IACUC guideline, and survival was recorded.

### Cluster analysis

8F4CAR-iNKT cells were assessed for marker expression via flow cytometry. Five thousand cells from each donor were analyzed using CRUSTY webtool where clusters were defined by Phenograph algorithm with default settings for UMAP generation.

### Statistical analysis

For *in vitro* and *in vivo* experiments, we used two-sided paired (for donor-matched comparisons) or unpaired (for inter-donor comparisons) Student’s t-test to evaluate statistical differences in variables between 2 groups. Analysis of Variance (ANOVA) was use to compare differences in variables among 3 or more groups. Survival was analyzed by the Kaplan-Meier method and Mantel-Cox test to compare groups. Statistics were measured with GraphPad Prism 10 software. Statistically significant differences were deemed as any P-value less than 0.05.

## Results

### Cord blood-derived iNK T Cells are exclusively CD4^+^ subtype with Th2/-10 polarized cytokine production profile, yet maintain potent cytolytic activity

**Figure 1:**
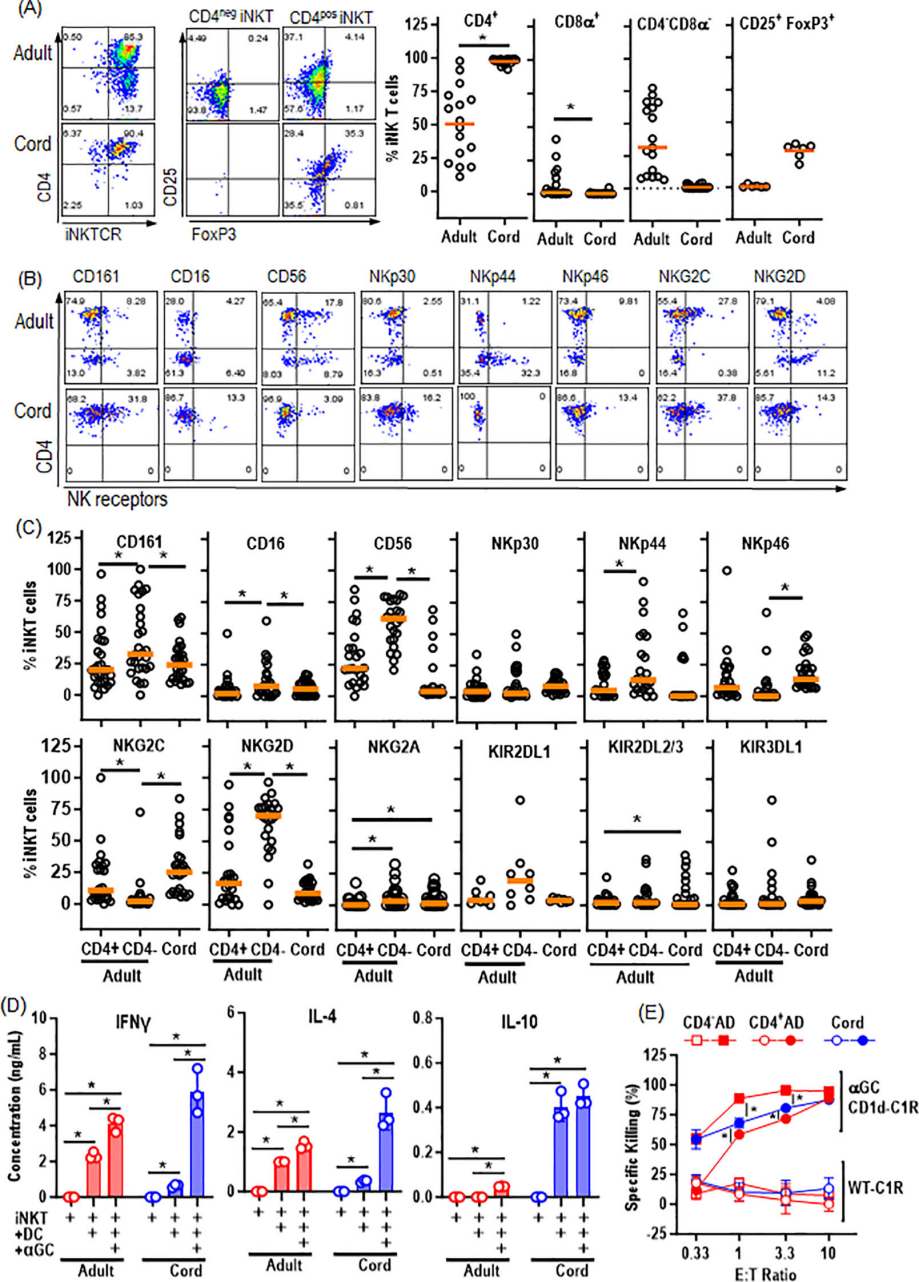
Cord blood-derived iNK T Cells are exclusively CD4^+^ subtype with Th2/-10 polarized cytokine production profile, yet maintain potent cytolytic activity. The iNKT cells were expanded from cord and adult donors, followed by phenotypic and functional assessment. **(A)** The representative phenotype analysis (left) and % expression of indicated markers on iNK T cells derived from cord blood (CB) and adult donor (AD)-iNKT cells. Each symbol represents a value from a single donor. **(B)** A representative expression of activating and inhibitory NK receptors on CB- and AB-iNKT cells. **(C)** The expression of indicated NK cell markers on CD4^+^ or CD4^-^ AB-iNKT cells, and CB-iNKT cells. Each symbol represents a value from a single donor. **(D)** The production of soluble IL-4, IL-10, and IFNγ by CB- and AB-iNKT cells after stimulation with dendritic cells (DC) +/- αGC for 72 hours. The experiment was performed in triplicates, and one of three independent experiments with unique iNKT donor. Mean +/- SD was represented. **(E)** Cytolysis of Target cells by CB or AD-iNK T cells. CD1d^+^ C1R cells were pulsed with αGC and incubated with indicated iNKT cells at indicated ratios of Effector to Target for 16hrs. Specific killing was calculated by counting the number of live target cells in experimental conditions against the number of live target cells without effector cells. Experiment was performed in triplicate, and one representative experiment of two independent experiments with different iNK T donors. Values were represented by Mean +/- SD. Unpaired student t-test was used to compare values between AD- and CB-iNK T cells or between different conditions. Paired student t-test was used to compare values between CD4^+^ vs CD4^-^ AD-iNK T cells derived from same donors. *, P < 0.05.

As we aim at evaluating cord blood-derived iNK T cells as offthe-shelf CAR T cell therapy platform, we first characterized short term, polyclonal iNK T cell lines established from AD and CB donors via single antigenic stimulation where magnetically sorted iNK T cells were expanded with αGalCer pulsed allogeneic dendritic cells (22, 23). First, the phenotype of CB-iNK T cells was exclusively CD4^+^ and enriched in potentially regulatory CD4^+^CD25^+^FoxP3^+^ iNK T cells, while AD-iNK T cells contained varying frequencies of CD4^+^ iNK T cells with large donor-to-donor variation and reciprocal portions of CD8α^-^CD4^-^ iNK T cells (**Figure 1A**). Interestingly, the expansion of highly inflammatory CD8α^+^ iNK T cells were observed only in adult donors. The higher expression of various activation NK receptors such as CD16/56, CD161, NKp44, and NKG2D were observed in CD4^-^ AD-iNK T cells than CD4^+^ AD- or CB-iNKT cells while CB-iNK T cells express NKp46 and NKG2C at higher levels than CD4^-^ AD-iNK T cells (**Figures 1B, C**). In contrast, there was a minimal expression of inhibitory NK receptors in both AD- and CB-iNK T cells (**Figures 1B, C**). Of note, the pattern of NKR expression of CB-iNKT cells were similar to those of CD4^+^ AD-iNKT cells but different from CD4^-^ AD-iNKT cells, indicating that observed the differences of NKR expression may be more attributable to the T cell subset (CD4^+^ vs CD4^-^) rather than the cellular origin (cord blood vs adult donor).

Upon antigenic stimulation with αGalCer pulsed dendritic cells, CB-iNK T cells were able to produce both Th1 (IFNγ), Th2 (IL-4), and Th10 (IL-10)-type cytokine in a Th2/10-polarized fashion compared to AD-iNK T cells (**Figure 1D**). Despite these immune-regulatory characteristics, CB-iNK T cells maintained antigen specific cytotoxic activity against αGalCer pulsed CD1d^+^ B-lymphoblastic leukemia cells (**Figure 1E**).

In summary, cord-blood derived iNK T Cells are enriched with CD4^+^CD25^+^FoxP3^+^NKR^low^ iNK T cells with Th-2/10 biased cytokine production profile upon iNK-TCR mediated activation, but maintain potent antigen specific cytolytic activities.

### Highly pure CAR-iNK T cells are reliably produced from the cord blood to a clinically meaningful number

**Figure 2:**
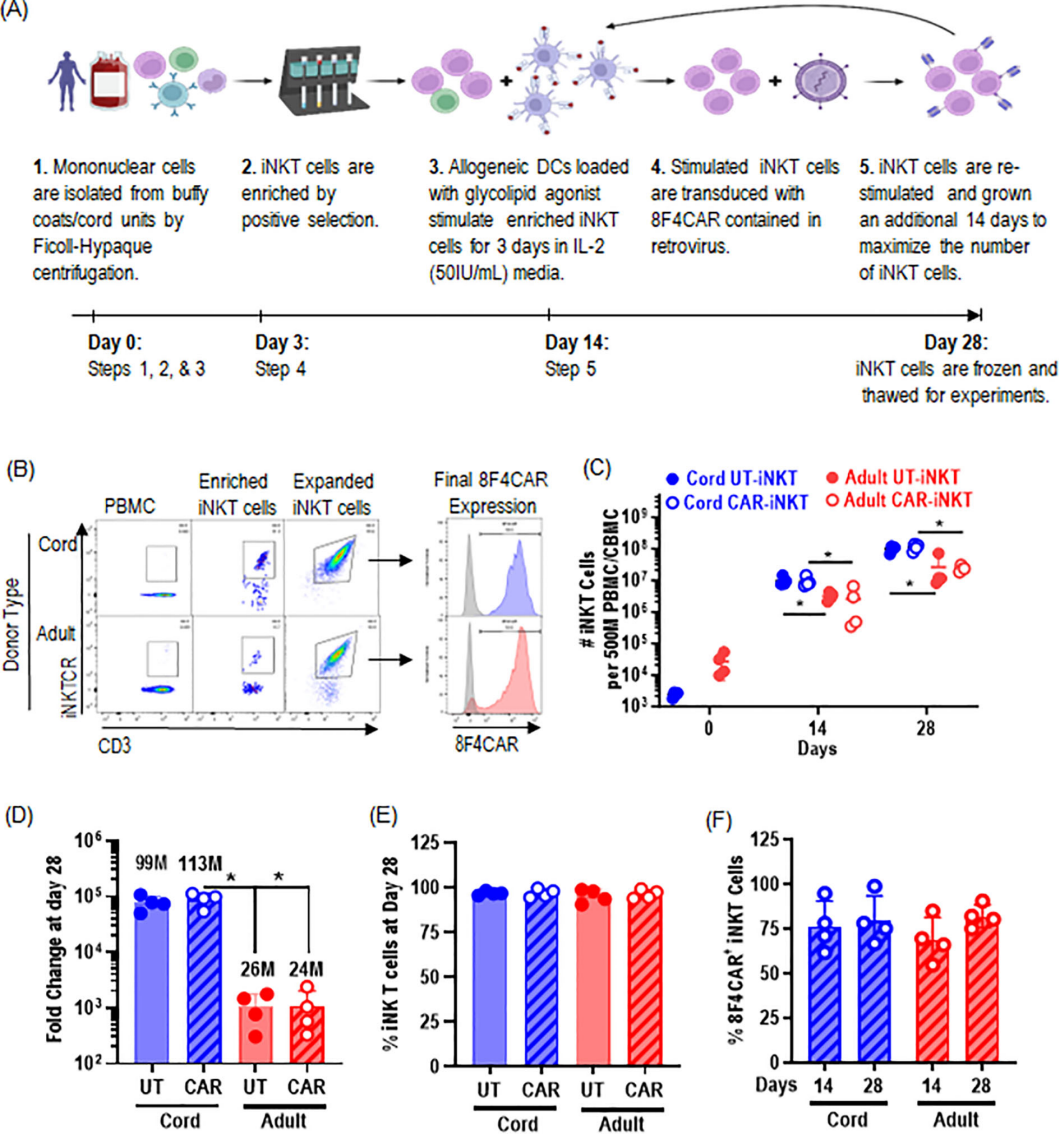
Highly pure CAR-iNK T cells are reliably produced from the cord blood to a clinically meaningful number. **(A)** Schematic of 8F4CAR-iNKT cell expansion protocol. The iNKT cells from cord blood units and adult peripheral blood were first isolated via magnetic cell sorting (MACS) with iNK-TCR-microbeads and stimulated by allogeneic dendritic cells pulsed with αGC. Activated iNK T cells were transduced with retrovirus harboring 8F4CAR and cultured for additional 14 days. Lastly, 8F4CAR-iNK T cells were re-expanded via single antigenic stimulation as described above. **(B)** Representative flow cytometry analysis of iNK T cells from cord and adult donor, in pre- and post-iNK T cell enrichment, and the final 8F4CAR expression (blue and red histogram indicate 8F4CAR-iNK T cells and grey is donor-matched UT-iNK T cells). **(C)** Absolute number of iNK T cells throughout the expansion protocol. **(D)** Total fold change of iNK T cells following the 28-day cell expansion protocol. Bolded numbers indicate the mean number of iNK T cells generated per 500M PBMC. Cells were counted by trypan blue exclusion. Mean +/- SD. ***, P < 0.001; ns, not significant; paired student t-test for intra-donor type comparison; unpaired student t-test for inter-donor type comparison. **(E)** iNK T cell purity of all live cells in culture determined by both CD3 and 6B11 positivity. Each point is a donor. **(F)** 8F4CAR expression at days 14 and 28 of the cell generation protocol by gating on 8F4CAR^+^ and 6B11^+^ iNKT cells. A symbol represents a value from a single donor. Unpaired student t-test was used to compare differences of values between CB vs AD-iNK T cells. Paired student t-test was used to compare differences of values between UT- or 8F4CAR-iNK T cells derived from the same donor. *, P < 0.05.

As we demonstrated that cord blood-derived iNK T cells maintain potent cytolytic activities despite Th2/Th10-polarized cytokine production profile, we investigated whether CAR-iNK T cells could be generated from the *ex vivo* isolated CB-iNK T cells using a modified expansion protocol to accommodate retroviral transduction of CAR construct (**Figure 2A**). First, iNK T cells were pulled from cord or peripheral blood mononuclear cells using iNK-TCR-microbeads per manufacturer’s instructions (**Figure 2B**), followed by antigenic stimulation with αGalCer-pulsed allogeneic dendritic cells as described previously (22, 23). After 72 hours, activated iNK T cells were transduced by retrovirus harboring 8F4CAR specific for PR1/HLA-A2 (21, 24), AML antigen, and cultured for additional 10 days. 8F4CAR-iNK T cells were subjected to second antigenic stimulation and final cell products were assessed for quality and quantity.

8F4CAR transduced iNK T cells were similar to untransduced (UT) iNK T cells in expansion capacity (fold of expansion and absolute number of iNK T cells after two consecutive antigenic stimulations) and iNK T cell purity (**Figures 2C-E**), supporting CAR-transduction did not impair the quality and quantity of iNK T expansion. Interestingly, CB-iNK T cells with or without 8F4CAR transduction expanded significantly better than AD-iNK T cells, with an average total fold expansion of ~89000 for CB-8F4CAR-iNK T and ~77000 for CB-UT-iNK T vs ~1100 for AD-8F4CAR-iNK T cells and ~1000 for AD-UT-iNK T cells leading to approximately 80-fold increase in expansion of CB-iNK T cells over AB-iNK T cells when normalized to the number of peripheral blood mononuclear cells (PBMCs) used as the starting material (**Figures 2C, D**). The iNK T cell purity of all cells alive in culture at the end of the cell generation protocol was an average of 96.89% vs 95.81% for CB- and AB- 8F4CAR iNK T cells, respectively (**Figure 2E**). 8F4CAR expression in CB-iNK T cells remained consistent at an average of 76.35% and 79.73% from day 14 to the final assessment at day 28 which is not statistically different from AD-iNK T cells (**Figure 2F**).

Therefore, CAR^+^ CB-iNK T cells can be reliably generated from ex vivo isolated cord iNK T cells in similar quality but in better expansion capacity to CAR^+^ AD-iNK T cells.

### CB-8F4CAR-iNK T cells are exclusively CD4^+^ and enriched with CD62L and ICOS

**Figure 3:**
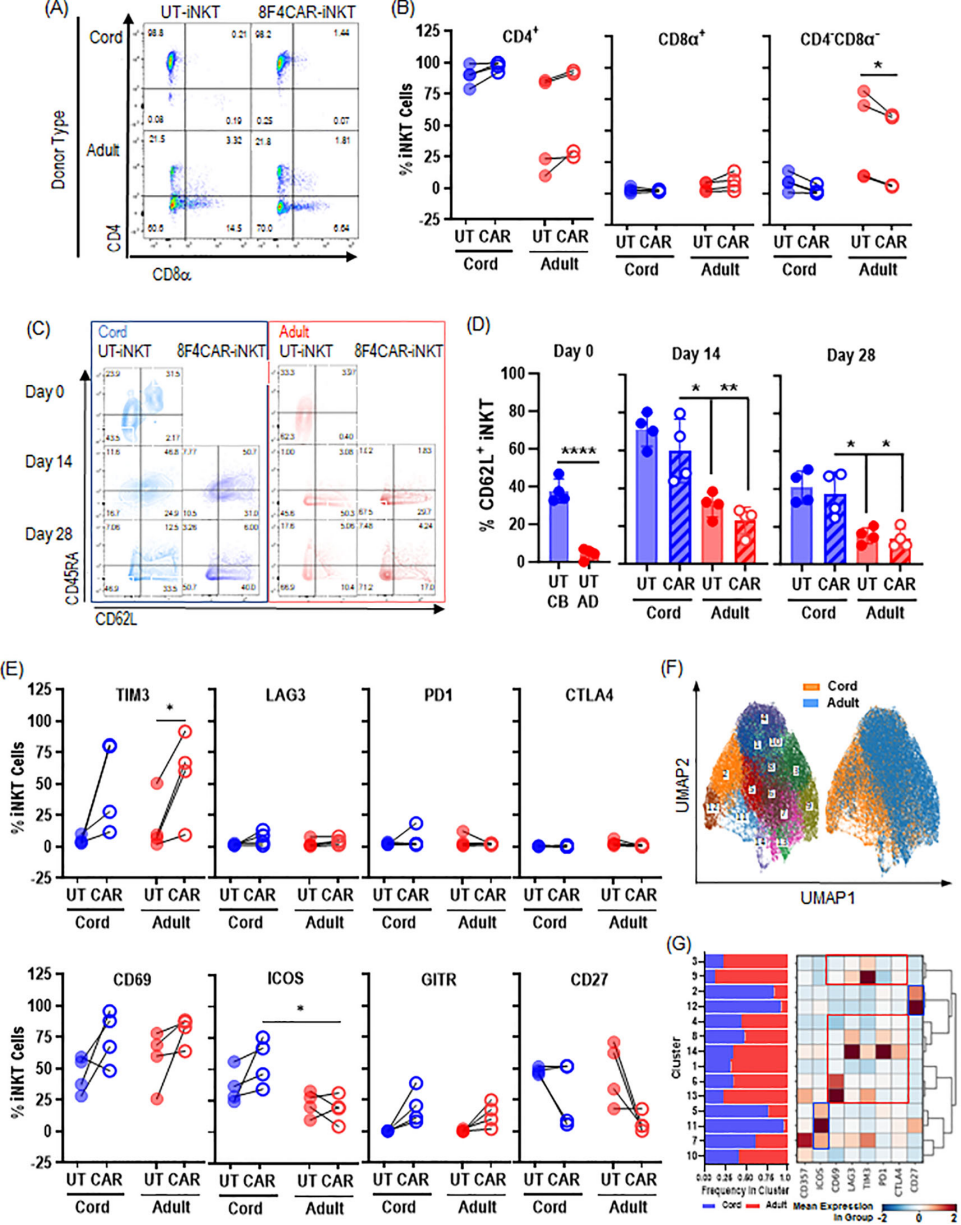
CB-8F4CAR-iNKT cells maintain a consistent CD4^+^ phenotype and are significantly enriched in CD62L^+^ and ICOS^+^ cells compared to AD-8F4CAR-iNKT cells. **(A)** A representative flow cytometric analysis of one cord and one adult donor UT- and 8F4CAR-iNK T cells. **(B)** The percentage of CD4^+^, CD8α^+^, and CD4^-^CD8α^-^ UT- and 8F4CAR-iNK T cells at the expansion protocol conclusion. *, P < 0.05; ns, not significant; paired student t-test for intra-donor type comparison. Each point is a donor. **(C)** Representative flow cytometric analysis of the memory phenotype, defined by CD62L and CD45RA, of UT- and 8F4CAR-iNK T cells from one cord and adult donor. **(D)** Percentage of CD62L^+^ iNK T cells throughout the cell expansion protocol. A symbol represents a value from a single donor. Mean +/- SD. *, P < 0.05, **, P < 0.01, ****, P < 0.0001; ns, not significant; paired student t-test for intra-donor type comparison; unpaired student t-test for inter-donor type comparison. **(E)** The percentage of activation and exhaustion markers on UT- and 8F4CAR-CB- and AD-iNK T cells determined by flow cytometry. Each point is a donor, *, P < 0.05; paired student t-test for intra-donor type comparison; unpaired student t-test for inter-donor type comparison. **(F)** Cluster analysis of 8F4CAR^+^ CB and AD-iNK T cells based on markers listed in panel E was done using open access software CRUSTY (39), showing segregation of CB-8F4CAR-iNK T cells from AD-8F4CAR-iNK T cells **(G)** The percentage of 8F4CAR^+^ CB and AD-iNK T cells in each cluster, and a heatmap cluster defining markers as determined by CRUSTY software, demonstrating that CB-8F4CAR-iNK T cells are enriched with ICOS^+^ or CD27^+^ clusters compared to AD-8F4CAR-iNK T cells.

The iNK T cells in adult peripheral blood are phenotypically heterogeneous compared to those in cord blood, with subsets generally defined by CD4 expression (**Figure 1A**) (18, 19). As expected, CB-iNK T cells were consistently CD4^+^ (range 79%-99%), whereas there was variation in CD4 expression in AB-iNK T cells (range 23.4%-85.6%). Interestingly, 8F4CAR transduction trended towards an increase in CD4^+^ cells and a subsequent decrease in CD4^-^ CD8a^-^ iNK T cells compared to donor-matched UT-iNK T cells for both donor types (**Figures 3A, B**). The CD62L^+^ naïve and central memory subtypes were significantly increased in *ex vivo* isolated CB-iNK T cells compare to AD-iNK T cells and persisted through two consecutive antigenic expansions. 8F4CAR transduction did not affect CD62L expression in CB-iNK T cells nor AD-iNK T cells. As the expression of CD62L on iNK T cells was shown to increase *in vivo* persistency of CAR-iNK T Cells (25), our results suggest that CB-8F4CAR-iNK T cells may display superior *in vivo* persistency than AD-8F4CAR-iNK T cells (**Figures 3C, D**).

To assess the fitness of 8F4CAR-iNK T cells after the expansion protocol we measured activation and exhaustion markers by flow cytometry. We found minimal expression of PD1, LAG3, and CTLA4 on both CB- and AD-8F4CAR-iNK T cells suggesting unimpaired anti-tumor function (**Figure 3E**). CB-8F4CAR-iNK T cells had significantly elevated ICOS expression relative to AD-8F4CAR-iNK T cells, with distinct ICOS-enriched populations identified in clusters 5, 11, and 7 by UMAP dimensionality reduction, suggesting enhanced anti-tumor potential of the CB-CAR-iNK T cells (**Figures 3F, G**) (26, 27).

### CB-8F4CAR-iNK T cells display effective lysis of PR1/HLA-A2^+^ leukemia *in vitro*

**Figure 4:**
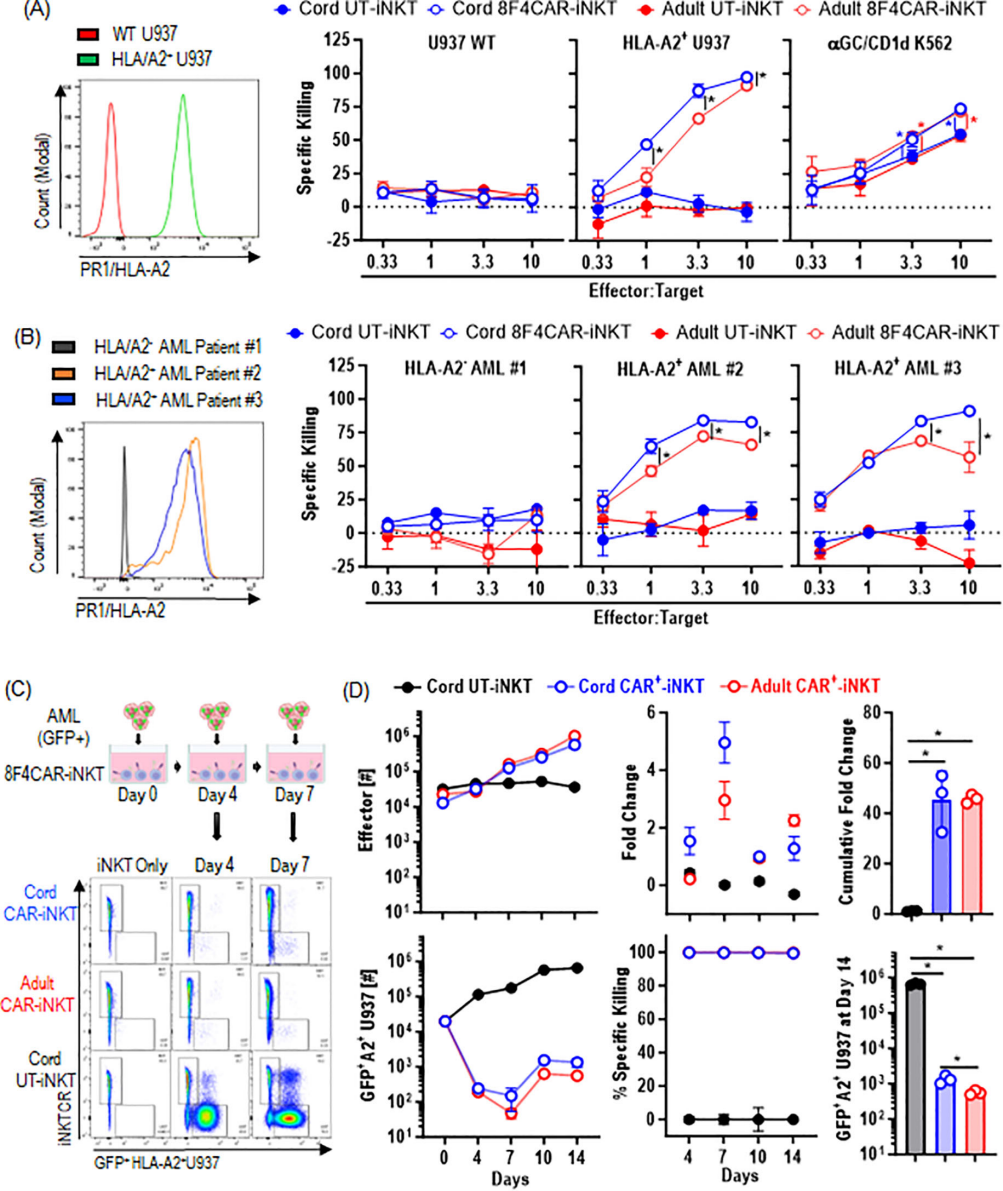
CB-8F4CAR-iNK T cells display effective lysis of PR1/HLA-A2^+^ leukemia *in vitro*. The indicated leukemia cell lines and primary AML patient samples were co-cultured with effector iNK T cells for 16 hours at indicated E:T ratios. Specific killing was calculated by counting the number of live target cells in experimental conditions against the number of live target cells without effector cells. **(A)** PR1/HLA-A2 expression on WT or HLA-A2^+^ U937 target cells used in cytotoxicity experiments (left panel), and specific killing of indicated target cells (right panel): WT U937, HLA-A2+ U937 cells, and αGC pulsed CD1d^+^ K562 cells by effector iNK T cells: CB-UT-iNK T cells, CB-8F4CAR-iNK T cells, AD-UT-iNK T cells and AD-8F4CAR-iNK T cells. One experiment from 3 independent experiments (each with a unique iNK T donor) was shown in triplicate, and represented with Mean +/- SD. **(B)** PR1/HLA-A2 expression on HLA-A2^-^ or HLA-A2^+^ AML primary cells obtained from three patients (left panel) and specific killing of indicated target cells by effector iNK T cells (right panel): CB-UT-iNK T cells, CB-8F4CAR-iNK T cells, AD-UT-iNK T cells and AD-8F4CAR-iNK T cells. One of two independent experiments were shown in triplicates and represented by Mean +/- SD. **(C)** A schematic of the repeat challenge cytotoxicity experiment (top panel); U937 HLA/A2^+^ GFP^+^ cells were co-cultured with effector cells at a 1:1 E:T ratio with 50 IU/mL IL-2. Every 3–4 days iNKT cells and target cells were counted and analyzed by flow cytometry and fresh U937 cells were added to maintain an E:T ratio of 1:1. A representative flow cytometric analysis of iNK T cells and remaining GFP+ HLA-A2^+^ U937 target cells throughout the experiment is shown (bottom panel). **(D)** Absolute numbers and fold of changes of effector iNK T cells after each round of leukemia cell challenge, and cumulated fold of change of iNK T cells at the conclusion of experiment. Absolute numbers of GFP+ HLA-A2^+^ U937 target cells after each round of leukemia cell challenge, and absolute number of GFP+ HLA-A2^+^ U937 target cells at the conclusion of experiment. One of two independent experiments with different iNK T donors was shown in Mean +/- SD, and experiment was performed in triplicates. Student t-test was used to compare values between groups. *; p<0.05.

Although human iNK T cells have overlapping functions, CD4^+^ iNK T cells are known to have lesser cytotoxic activity compared to CD4^-^ iNK T cells (18, 28). As CB-8F4CAR-iNK T cells are predominantly CD4^+^, we carefully evaluated their cytolytic activity against PR1/HLA-A2^+^ or αGalCer/CD1d^+^ leukemia and compared those with AD-8F4CAR-iNK T cells derived from multiple donors. To investigate the cytolytic activity of 8F4CAR-iNK T cells mediated by the 8F4CAR and the iNK-TCR, we co-cultured the effector iNK T cells with PR1/HLA-A2**^+^** or αGalCer/CD1d**^+^** leukemia cell lines (**Figure 4A**), and PR1/HLA-A2**^+^** primary AML patient samples (**Figure 4B**), then assessed the specific killing of target cells. First, there was no significant killing of PR1/HLA-A2^-^ wild-type U937 or primary AML cells when incubated with UT- or 8F4CAR-iNK T cells derived from both AD and CB donors. Interestingly, CB-8F4CAR-iNK T cells exhibited superior cytolysis against PR1/HLA-A2^+^ U937 cells and primary AML patient samples compared to AD-8F4CAR-iNK T cells, while CB-8F4CAR-iNK T cells retained the ability to lyse CD1d^+^ cell lines at a similar level compared to AD-8F4CAR-iNK T cells (**Figures 4A, B**). Notably, both AD- and CB-8F4CAR-iNK T cells consistently showed better iNK-TCR mediated lytic activities compared to UT counterparts, suggesting that 8F4CAR transduction may have increased the cytolytic machinery of iNK T cells (**Figure 4A**).

To assess the functional fitness of CB-8F4CAR-iNK T cells, we conducted repeated leukemia challenge assays. In this assay, we co-cultured effector cells with GFP-expressing U937 HLA-A2^+^ cells for 4 days, measured the number of live leukemia targets and live effector cells to calculate proliferation of effector iNK T cells and their cytolytic activity, and then added fresh leukemia cells at a ratio of 1 effector to 1 target. This process was repeated for 4–5 cycles (**Figure 4C**). First, both CB- and AD-8F4CAR-iNK T cells showed consistent and efficient anti-leukemic activities throughout serial antigenic challenges, and although CB-8F4CAR-iNK T cells showed a lesser CTL activity than AD 8F4CAR iNK T cells in terms of absolute number of remaining leukemia target cells, there was a statistically non-significant difference in specific killing (average 99.7% vs 99.5% at 4^th^ leukemic challenges) (**Figure 4D**). As expected from higher percentage of CD62L^+^ iNK T subsets, we observed a trend where CB-8F4CAR-iNK T cells expanded in greater number compared to AD-8F4CAR-iNK T cells with earlier leukemic challenges (**Figure 4D**). Thus, the results suggest the cytolytic potential of CB-iNK T cells can be successfully re-directed with antigen specific CAR, and that CB-CAR-iNK T cells were not inferior in their *in vitro* CTL activities compared to AD-CAR-iNK T cells.

### CB-8F4CAR-iNK T cells significantly decrease leukemia burden *in vivo* and extend survival of xenogeneic AML mice

**Figure 5:**
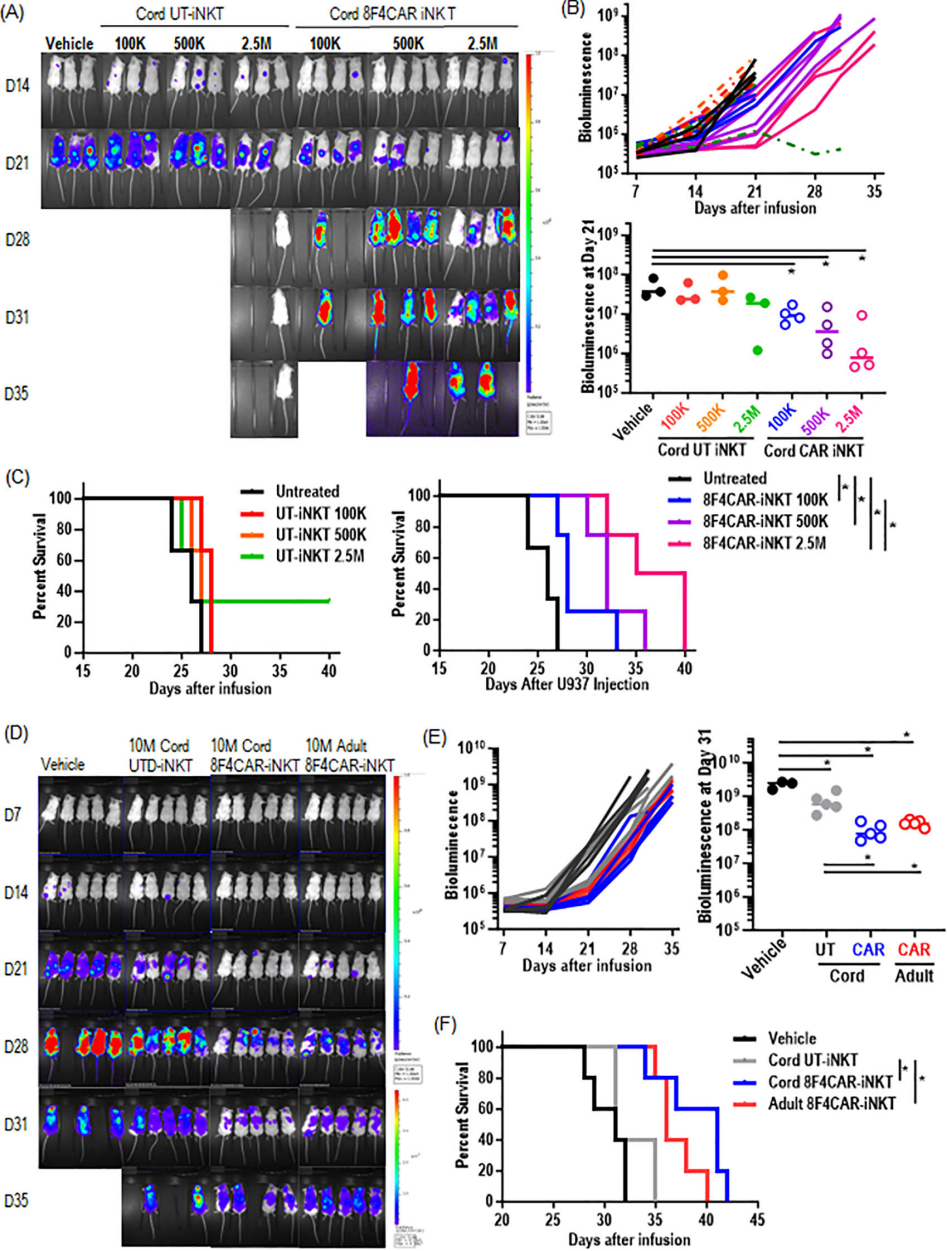
CB-8F4CAR-iNK T cells significantly decrease leukemia burden *in vivo* and extend survival of xenogeneic AML mice. NSG mice were intravenously injected with HLA/A2+luciferase^+^ U937 cells via tail vein followed by UT- or 8F4CAR-iNK T cells three days later. Leukemia progression was monitored by weekly bioluminescence imaging (BLI). **(A)** BLI images beginning at day 14 post leukemia cell injection for CB-8F4CAR-iNK T cells or CB-UT-iNK T cells in different doses. One mouse in the 2.5M CB-UT-iNK T cell group did not show successful leukemia engraftment. **(B)** BLI signal at the last time point where all mice were alive for mice depicted in panel **(A)**. **(C)** Survival curves of the xenografted mice treated with UT- or 8F4CAR-CB-iNK T cells. **(D-F)** NSG mice were intravenously injected with HLA/A2^+^ luciferase^+^ U937 cells followed by UT- or CB-8F4CAR-iNK T cells or AD-8F4CAR-iNK T cells at a dose of 10e7 three days later. Leukemia progression was monitored by weekly BLI. **(D)** BLI images **(D)**, BLI intensity **(E)**, and percent survival **(F)** of xenografted mice treated with CB-UT-iNK T cells, CB-8F4CAR-iNK T cells or AD-8F4CAR-iNK T cells. Student t-test was used to compare differences between group, and Log-rank Mantel-Cox statistical assessment was used to calculate the differences of survival between groups. A symbol represents a value from a mouse. *, P < 0.05.

As CB-8F4CAR-iNK T cells are shown to maintain efficient anti-leukemic activity *in vitro*, we next evaluated the ability of CB-8F4CAR-iNK T cells to control leukemia growth in a xenogeneic AML model. NOD scid gamma (NSG) mice were intravenously injected with U937 HLA-A2^+^ luciferase-expressing cells on day 0, followed by intravenous treatment of CB-8F4CAR-iNK T cells or donor-matched UT-CB-iNK T cells as a control, three days later. We found that mice treated with CB-8F4CAR-iNK T cells had significantly decreased leukemia burden compared to mice treated with CB-UT-iNK T cells in a dose dependent manner (**Figures 5A, B**), which lead to the significant survival benefit of mice treated with CB-8F4CAR-iNK T cells compared those treated with control groups, trending in a dose-dependent manner (**Figure 5C**). Further, mice treated with CB-8F4CAR-iNK T cells exhibited similar leukemia reduction and survival benefit compared to mice treated with AD-8F4CAR-iNK T cells at the same dose (**Figures 5D-F**). There were no visible indications of graft-versus-host disease-like symptoms observed, and mouse weight remained stable in all groups (data not shown).

In summary, CB-8F4CAR-iNK T cells demonstrated efficient dose-dependent *in vivo* anti-leukemic activity and non-inferiority compared to AD-8F4CAR-iNK T cells.

### CB-8F4CAR-iNK T Cells maintain Th2/10-biased cytokine production profile upon iNK-TCR and 8F4CAR mediated activation

**Figure 6:**
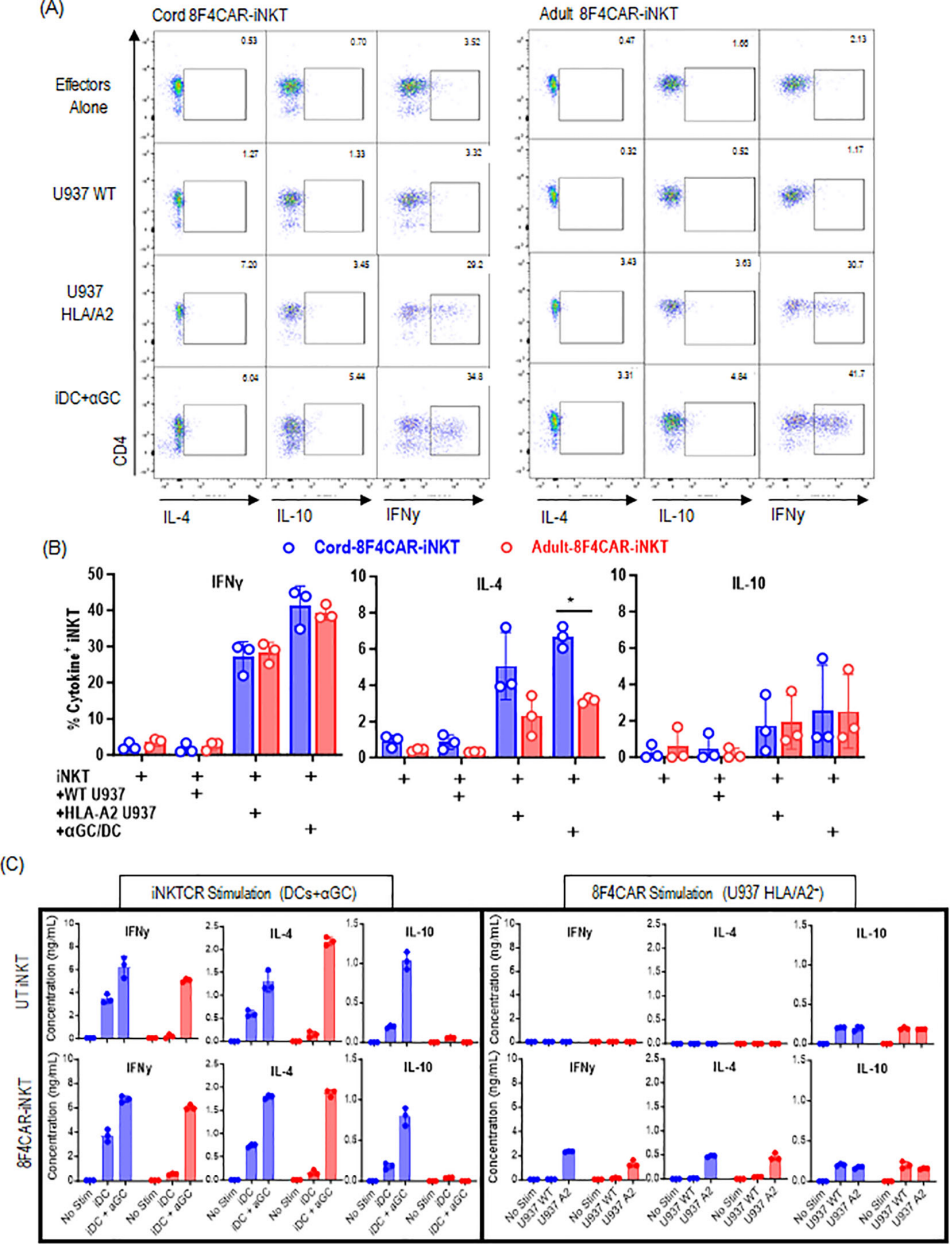
CB-8F4CAR-iNK T Cells maintain Th2/10-biased cytokine production profile upon iNK-TCR and 8F4CAR mediated activation. CB or AD-8F4CAR-iNK T cells were co-cultured with various target cells (WT-U937, HLA-A2^+^ U937, αGC/DC) for 8 hours at a ratio of 1 effector to target and subsequently stained for intracellular cytokines for flow cytometry analysis. **(A)** Representative flow cytometric analysis of intracellular IL-4, IL-10, and IFNγ production by effector iNK T cells after antigenic stimulation. **(B)** Percent IL-4^+^, IL-10^+^, and IFNγ^+^ CB- and AD-8F4CAR-iNK T cells after antigenic stimulation. **(C)** CB- or AD-8F4CAR iNK T cells were co-cultured with target cells (WT-U937, HLA-A2^+^ U937, DC, αGC/DC) for 24 hours, and culture supernatants were analyzed for soluble cytokine production. Experiments were performed in triplicates, and one of three independent experiments with unique iNK T donor was shown in mean +/- SD. Student t-test was used to compare values between groups. *; p < 0.05.

One potential advantage of CB-iNK T cells over AD-iNK T cells as the candidate for off the shelf cell therapy is their potential to regulate cytokine release syndrome given their ability to produce IL-10 and the enrichment of CD4^+^FoxP3^+^ cells (**Figure 1D**). Here, we investigated whether CB-8F4CAR-iNK T cells maintain the ability to produce cytokines in Th2/10-biased fashion upon iNK-TCR and 8F4CAR-mediated activation. UT or 8F4CAR-iNK T cells from AD and CB donors were stimulated by PR1/HLA-A2^+^ U937 cells (activation via 8F4CAR) or αGalCer/DC (activation via iNK-TCR), and intracellular (**Figures 6A, B**) or soluble cytokines (**Figure 6C**) were assessed 8 hours or 24 hours post stimulation, respectively. First, 8F4CAR-iNK T cells from both AD and CB donors produced both soluble Th1 and Th2/10-type cytokine similarly to those from donor-matched UT-iNK T cells upon αGalCer/DC-iNK-TCR mediated stimulation. Furthermore, CB-8F4CAR-iNK T cells maintained Th2/10-biased cytokine production profile (**Figure 6C**). However, 8F4CAR-iNK T cells showed a significantly less cytokine production, especially IFNγ, upon PR1/HLA-A2-8F4CAR mediated activation compared to αGalCer/CD1d-iNK-TCR mediated activation (**Figures 6B, C**) despite the efficient cytolytic activity. Thus, our results demonstrated that CB-8F4CAR-iNK T cells maintain Th2/10-polarized cytokine production upon iNK-TCR mediated activation, while significantly less inflammatory cytokine production upon 8F4CAR-mediated stimulation, supporting the dual function as 8F4CAR mediated effector and iNK-TCR mediated regulator.

## Discussion

CAR-T cell therapy represents a promising approach for treating hematopoietic malignancies and solid tumors. The use of allogeneic CAR-T cells is currently being investigated to overcome obstacles of autologous CAR-T cells such as a prolonged production time, high production costs, and potential manufacturing inconsistencies and potential failure (29–31). As CD1d-restricted invariant Natural Killer (iNK) T cells are a suitable candidate for allogeneic CAR-T cell therapy, we aimed at investigating iNK T derived from cord blood as a novel platform for allogeneic CAR-T cell therapy.

Our research reveals several key advantages of cord blood-derived iNK T cells that position them as a promising off-the-shelf CAR-T cell therapy platform. First, we observed significant inter-donor homogeneity in CB-iNK T cells, in contrast to the phenotypic heterogeneity observed in adult donor-derived iNK T cells. While adult-derived iNK T cells demonstrated significant variability in CD4 and NK receptor expression, CB-iNK T cells were consistently CD4+ (79-99%), with a uniform phenotype that could potentially translate to more consistent therapeutic products.

The expansion capacity of CB-iNK T cells is particularly noteworthy. Our findings demonstrate an approximately 80-fold better expansion compared to adult-derived iNK T cells and significantly higher number of the final cell product when normalized to the number of peripheral or cord blood mononuclear cells used as the starting material. It is likely that the increased proliferation of CB-iNK T cells is due to increased CD62L expression, indicating an enrichment in naïve/central memory populations, which is also associated with increased *in vivo* persistence of CAR-iNK T cells (25). This substantial expansion potential is crucial for generating clinically meaningful cell numbers, a critical consideration for cell-based therapies. Moreover, the CAR transduction process did not compromise the expansion capacity or purity of the iNK T cells, with final product purity consistently around 96%. As the expansion of CAR-T cells in patients is a predictor of efficacy in treating hematopoietic malignancies (32, 33), we posit that CB-iNK T cells may be advantageous over AD-iNK T cells.

The enrichment of CD4^+^CD25^+^FoxP3^+^ cells in CB-iNK T cells presents a unique immunomodulatory mechanism that could potentially mitigate the risks associated with cytokine release syndrome (CRS). This phenotype is similar to regulatory T cells (Tregs) that have been extensively studied for their immunosuppressive properties (34, 35). These cells can suppress the activation and proliferation of effector T cells through multiple mechanisms, including the secretion of anti-inflammatory cytokines like IL-10 and TGF-β (36, 37). In the context of CAR-T cell therapies, this suppressive capacity could be particularly valuable in managing potentially life-threatening CRS. The enrichment of CD4^+^CD25^+^FoxP3^+^ iNK T cells in CB donors suggests a potential protective role against CRS. Their ability to produce IL-10 and maintain a Th2/Th10-polarized cytokine profile suggests a natural mechanism for dampening inflammatory responses and warrants further study.

The *in vitro* and *in vivo* efficacy of CB-8F4CAR-iNK T cells against PR1/HLA-A2^+^ leukemia cells was particularly promising. Despite the predominance of Th2/Th10-biased CD4^+^ iNK T cells, which are traditionally associated with lower cytotoxic potential, CB-8F4CAR-iNK T cells demonstrated non-inferiorlysis of leukemia targets compared to adult-derived counterparts. The repeated leukemia challenge assays further validated their consistent anti-leukemic activities, with nearly identical specific killing rates across multiple challenges. Further, the xenogeneic AML mouse model provided compelling evidence of the therapeutic potential of CB-8F4CAR-iNK T cells. Treatment with these cells significantly decreased leukemia burden and extended survival in a dose-dependent manner, comparable to AD-8F4CAR-iNK T cells. Notably, no graft-versus-host disease (GvHD)-like symptoms were observed, supporting the inherent advantage of iNK T cells restricted to the monomorphic CD1d protein (8, 9).

Additionally, emerging evidence has demonstrated that ICOS expression in CAR-T cells correlates with superior anti-tumor efficacy. Recent studies have shown that ICOS^+^ CAR-T cells exhibit enhanced persistence, improved cytokine production, and more robust tumor cell killing (26, 27). Furthermore, engineering CAR-T cells with ICOS-based costimulatory domains has been shown to augment their anti-tumor activity, particularly against solid tumors where traditional CAR-T approaches have shown limited success (38). The critical role of ICOS signaling in maintaining T cell function and survival within the immunosuppressive tumor microenvironment has positioned it as a promising target for next-generation CAR-T cell design. Interestingly, CB-8F4CAR-iNK T cells expressed higher levels of ICOS compared to AD-8F4CAR-iNK T cells which may attribute in part to the efficientanti-leukemic activity. These characteristics, combined with the cells’ robust expansion capacity and controlled inflammatory response, positions CB-iNK T cells as an attractive platform for allogeneic CAR T cell therapy.

In conclusion, our study provides evidence that CB-CAR-iNK T cells could be a valid alternative option to AD-CAR-iNK T cells for the development of an allogeneic off-the shelf therapy. By leveraging the unique properties of CB-iNK T cells and the tumor specificity of the CAR, we believe that CB-CAR iNK T cells will serve a more accessible, effective, and safe treatment option for patients with hematologic malignancies and solid tumors.

## Data Availability

The original contributions presented in the study are included in the article/supplementary material. Further inquiries can be directed to the corresponding authors.
